# Delirium Tremens Successfully Treated with Coffee

**Published:** 1867-12

**Authors:** Wm. R. Whitehead

**Affiliations:** New York


### DELIRIUM TREMENS SUCCESSFULLY TREATED WITH COFFEE.

By WM. R. WHITEHEAD, M.D., New York.

Recently, in a case of delirium tremens, I observed a peculiarly marked tranquillizing effect caused by strong coffee, and which produced prolonged and refreshing sleep, after the usual remedies had proved ineffectual.

Mr. C., a merchant, visiting New York on business, who had been drinking very hard for several days, was taken with delirium tremens on the 13th of last March. His friends stated that they believed this to be his first attack. He was seen by a physician on the 15th, but did not receive further medical attention until the night of the 17th, when I was called to see him. He exhibited the usual symptoms, with the exception of a remarkable absence of muscular twitching on feeling the pulse. His hallucinations were at times very amusing; he was not disposed to be violent, but was quite tractable and easily controlled by his attendants. A mixture containing about a drachm of chloroform to the dose, was prescribed to be taken every few hours.

The next morning, he was more quiet, having slept about two hours during the night, and for the first time since his illness. The condition of his bowels, his urinary and other secretions, were carefully observed. His tongue was large, white, and moist. Some pills of opium were substituted for a mixture containing chloroform and tincture of hops, ordered early in the day. A small quantity of ale was permitted, and some beef-tea and chicken-broth ordered. At night, he was very restless; his pulse at one time could not be felt; his extremities became cold and respiration labored. I gave him half a glass of whiskey.

On the morning of the 19th, I was told that he slept an hour or two soon after drinking the whiskey; his skin was jaundiced, and he was costive. Twelve grains of blue mass were prescribed, which failed to produce the desired effect. At night, eight grains of calomel were given, to be followed in the morning by a clyster. He vomited several times during the day, but retained the nourishment which he had taken.

On the 20th, his bowels were slightly relieved, his skin was moist, and pulse good. I prescribed sulphate of morphia, in half-grain doses; he became quite restless during the day.

The next day, there was no sensible abatement of the restlessness, and he was not free for any length of time from ludicrous or disquieting hallucinations. At no time, however, was he violent, nor did he manifest great fright. The sulphate of morphia was repeated; he took some nourishment; his bowels were moved with a clyster. Complaining of soreness in the stomach; a blister was placed on the epigastrium.

On the 22d, at the morning visit, being satisfied that the morphia had failed to afford relief; bromide of potassium was given, twenty grains at a dose, every two hours, in a suitable mixture. At the third dose, he became more restless and excited than he had previously been. The bromide was discontinued, and three subcutaneous injections of Magendie's solution of morphia were made, of thirty drops each, at an interval of one hour between each injection. They failed to produce the slightest apparent effect. He drank half a glass of whiskey, which seemed to quiet him considerably. He took frequently during the day Tourtelot's beef-essence with much relish.

On the morning of the 23d, I was told that after drinking the whiskey, he slept three hours, but I found him quite restless. Bromide of potassium was prescribed, in drachm doses, of which he took two, each at an interval of two hours. At half-past four P.M., he was much more quiet. He asked for a cup of coffee, which he drank very gratefully, and at the same time ate a small piece of bread. Ninety grains of bromide of potassium were ordered at a dose, in which doses, I was reliably informed, it had been recently given successfully in delirium tremens at the New York Hospital. During the night, two doses of the bromide were administered. His urine was abundant and clear; pulse strong and full. He slept about an hour that night. The next day, his bowels were slightly moved with a clyster. The bromide of potassium was repeated twice, in doses of ninety grains, and produced at the second dose some anæsthesia of the skin.

On the 25th, Prof. Willard Parker saw him with me, and observed the same absence of muscular twitching to which I have already alluded. It was decided that the patient should have his body and limbs well rubbed after being washed; that he should be permitted to have a cup of coffee, and as much nourishment as he could be induced to take. At night, a hot whiskey toddy was to be given to him, to be succeeded by inhalations of ether, but the ether was not given.

At the morning visit the next day, I found that he had not slept during the night, and was very restless. The toddy seemed at first, as his nurse informed me, to make him more quiet; but he soon became much more restless after than before taking it. Chloroform, opium, brandy, and bromide of potassium had been each unsuccessfully essayed; the last of these substances in such doses as thoroughly to test its action. One prominent idea, however, governed my course throughout the treatment—it was properly to nourish the patient. I regarded the alcohol as a poison to be eliminated, but considered that the equilibrium of the nervous system should be gradually reëstablished by suitable stimulants. Only temporary benefit resulted from the remedies used; and if at times they failed to produce any appreciable good, they, on the contrary, once or twice seemed to be productive of harm. When quiet and sleep did not immediately follow the use of brandy, the restlessness was much increased. Morphia appeared to be positively injurious. The bromide of potassium, in doses of twenty grains, greatly excited the patient; increased to drachm doses, and then to doses of one drachm and a-half each, made him a little more quiet. I was not encouraged, however, to continue any longer these remedies, but disposed to discontinue further medical treatment, and rely upon the gradual assimilation of concentrated and nutritious food, to allay the nervous excitement, and produce sufficient recuperative sleep.

The patient had, several days before, expressed a wish for coffee, which he seemed to relish. It now occurred to me that coffee, acting as a food, containing nitrogenous principles, and also as a special nervous stimulant, might in this case, by its peculiar action, if given in sufficient quantities, produce a quieting effect, and induce sleep. Consequently, coffee was tried; and the issue of the experiment proved to be successful.

At two o'clock P.M., about a pint of very strong coffee was prescribed, with a little cream, enough to make it palatable for him; and a broiled steak ordered, a part of which he ate. At eight o'clock P.M., I found that he had slept four hours. He drank again a large cup of coffee, and took fifteen grains of blue mass.

On the 27th, at the morning visit, I found that he had slept nearly all night, but was then restless. His bowels had been moved, and his general condition was very much improved. Very strong coffee was again prescribed, and at half-past twelve o'clock P.M., I found him asleep. After this, the coffee was repeated a few times; he continued to improve, and soon recovered.

Probably this nervous stimulant, by imparting an increased tone of a peculiar character to the general system, reëstablished its equilibrium and caused sleep, when other stimulants, acting in a different manner, failed to produce this result. This, however, is merely a conjecture, and may possibly be explained by others in a more satisfactory manner.—*Medical Record.*

